# Supplementary material for: Transcriptome Analysis and Functional Characterization of the HvLRR_8-1 Gene Involved in Barley Resistance to Pyrenophora graminea
Source: Plants (Basel). 2025 Jul 30;14(15):2350. doi: 10.3390/plants14152350 (PMC12348818; doi:10.3390/plants14152350)
Supplement: Supplementary file 1 [file plants-14-02350-s001.zip › Supplementary Figure.docx]

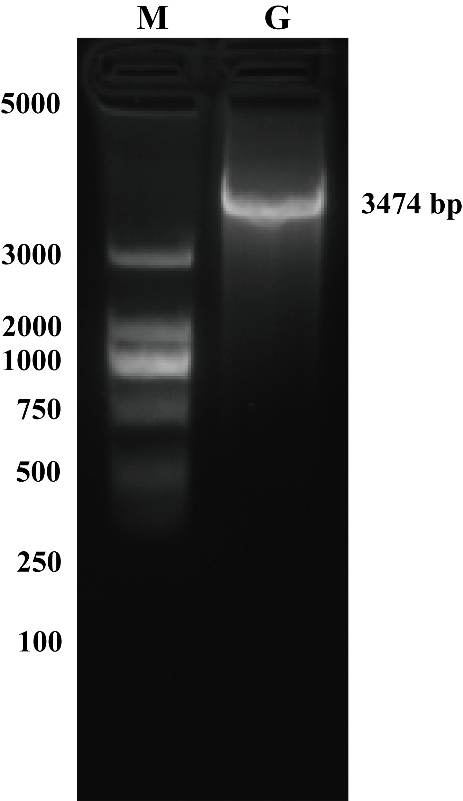


**Supplementary Figure 1 Gel electrophoresis of *HvLRR_8-1* gene clone.**

M: 5000 bp Marker, G:*HvLRR_8-1* gene


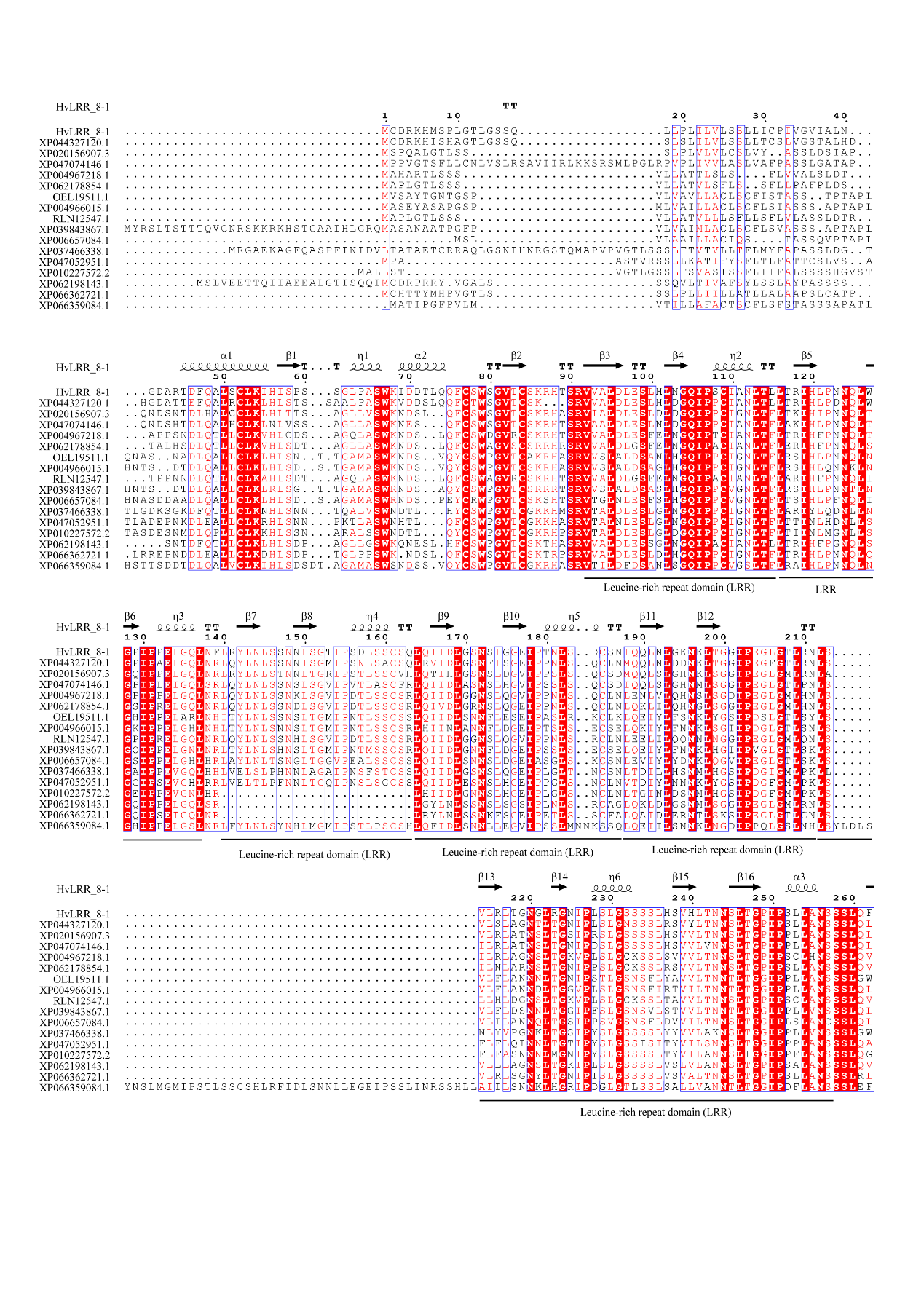


**Supplementary Figure 2 Alignment diagram of barley *HvLRR_8-1* with** **sequences containing LRR domain from different species.**
