# Supplementary material for: Transcriptome Analysis and Functional Characterization of the HvLRR_8-1 Gene Involved in Barley Resistance to Pyrenophora graminea
Source: Plants (Basel). 2025 Jul 30;14(15):2350. doi: 10.3390/plants14152350 (PMC12348818; doi:10.3390/plants14152350)
Supplement: Supplementary file 1 [file plants-14-02350-s001.zip › Supplementary Table/Table S18 The Neighbor-Joining (NJ) phylogenetic tree was constructed using MEGA 11 the resulting “Newick” tree file.docx]

(((((((((((XP_039843867.1_Panicum_virgatum,KAG2612508.1_Panicum_virgatum)1.0000,XP_025810626.1_Panicum_hallii)0.9960,XP_004966015.1_Setaria_italica)1.0000,OEL19511.1_Dichanthelium_oligosanthes)0.7300,XP_066359084.1_Miscanthus_floridulus)0.7150,TVT96651.1_Eragrostis_curvula)1.0000,(XP_006657084.1_Oryza_brachyantha,(KAF2927321.1_Oryza_sativa,XP_052159487.1_Oryza_glaberrima)1.0000)1.0000)1.0000,(XP_015641486.1_Oryza_sativa,((XP_037466338.1_Triticum_dicoccoides,XP_044423367.1_Triticum_aestivum)1.0000,(XP_047052951.1_Lolium_rigidum,(XP_047074161.1_Lolium_rigidum,XP_010227572.2_Brachypodium_distachyon)0.9630)0.6770)1.0000)1.0000)0.9400,((XP_004979262.1_Setaria_italica,XP_062198143.1_Phragmites_australis)1.0000,(XP_062178854.1_Phragmites_australis,(XP_004967218.1_Setaria_italica,(RLN12547.1_Panicum_miliaceum,PUZ60158.1_Panicum_hallii)1.0000)1.0000)1.0000)0.5980)0.8560,(XP_002440678.1_Sorghum_bicolor,(XP_066362721.1_Miscanthus_floridulus,XP_066362718.1_Miscanthus_floridulus)1.0000)1.0000)0.9860,((XP_037468204.1_Triticum_dicoccoides,XP_037468895.1_Triticum_dicoccoides)1.0000,((KAK1610963.1_Lolium_multiflorum,XP_047074146.1_Lolium_rigidum)1.0000,((XP_044435952.1_Triticum_aestivum,XP_037463734.1_Triticum_dicoccoides)1.0000,(XP_048541431.1_Triticum_urartu,XP_020156907.3_Aegilops_tauschii)0.9930)1.0000)0.9990)0.9990,((HvLRR_8-1_Hordeum_vulgare,XP_044970939.1_Hordeum_vulgare)1.0000,(((KAE8812944.1_Hordeum_vulgare,XP_044971101.1_Hordeum_vulgare)1.0000,XP_037488104.1_Triticum_dicoccoides)0.9910,((KAE8812949.1_Hordeum_vulgare,XP_044459957.1_Triticum_aestivum)1.0000,(XP_048553776.1_Triticum_urartu,(XP_044327120.1_Triticum_aestivum,XP_044335824.1_Triticum_aestivum)1.0000)1.0000)0.6060)0.5380)1.0000);
